# Time-Dependent Prognostic Impact of Circulating Tumor Cells Detection in Non-Metastatic Breast Cancer: 70-Month Analysis of the REMAGUS02 Study

**DOI:** 10.1155/2013/130470

**Published:** 2013-05-09

**Authors:** François-Clément Bidard, Lisa Belin, Suzette Delaloge, Florence Lerebours, Charlotte Ngo, Fabien Reyal, Séverine Alran, Sylvie Giacchetti, Michel Marty, Ronald Lebofsky, Jean-Yves Pierga

**Affiliations:** ^1^Department of Medical Oncology, Institut Curie, 26 rue d'Ulm, 75005 Paris, France; ^2^Laboratory of Circulating Cancer Biomarker, Institut Curie, 26 rue d'Ulm, 75005 Paris, France; ^3^Department of Biostatistics, Institut Curie, 26 rue d'Ulm, 75005 Paris, France; ^4^Department of Medicine, Institut Gustave Roussy, 39 rue Desmoulins, 94805 Villejuif, France; ^5^Department of Surgery, Institut Curie, 26 rue d'Ulm, 75005 Paris, France; ^6^Department of Medical Oncology, Hôpital Saint Louis, 1 Avenue Vellefaux, 75011 Paris, France; ^7^Université Paris Descartes, 15 rue de l'École de Médecine, 75006 Paris, France

## Abstract

*Introduction*. In non-metastatic breast cancer patients, the REMAGUS02 neoadjuvant study was the first to report a significant impact of circulating tumor cells (CTCs) detection by the CellSearch system on the distant metastasis-free survival (DMFS) and overall survival (OS) endpoints. However, these results were only reported after a short follow-up. Here, we present the updated data, with a longer follow-up. *Material and Methods*. CTC count was performed before and after neoadjuvant chemotherapy in 118 patients and correlated to survival. *Results*. CTC count results were available before and/or after neoadjuvant chemotherapy in 115 patients. After a median follow-up of 70 months, detection of ≥1 CTC/7.5 mL before chemotherapy (*N* = 95) was significantly associated with DMFS (*P* = 0.04) and OS (*P* = 0.03), whereas postchemotherapy CTC detection (*N* = 85) had no significant impact. In multivariable analysis, prechemotherapy CTC and triple negative phenotype were the two independent prognostic factors for survival. We observed that the CTC impact is most significant during the first three years of follow-up. *Discussion*. We confirm that the detection of CTC is independently associated with a significantly worse outcome, but mainly during the first 3-4 years of follow-up. No prognostic impact is seen in patients who are still relapse-free at this moment.

## 1. Introduction

Over the past decade, two opportunities have been created to study the hematogenous metastatic process in breast cancer patients: disseminated tumor cells (DTCs), which are detected in the bone marrow, and circulating tumors cells (CTCs), which are detected in the blood [1]. In non-metastatic breast cancer patients, a pooled analysis published in 2005 established that bone marrow DTC detection is an independent prognostic factor of later metastatic relapse [2]. Survival data obtained in this analysis, as well as in other studies [3, 4], were based on a long follow-up, generally of more than 5 years, and suggested that the prognostic impact of DTC (i.e., hazard ratio of relapse or death) was rather constant over time in the population at risk. This finding is consistent with the fact that DTCs are quiescent tumor cells and can stay in G0 phase for many years, before metastatic growth.

In contrast, CTCs have been mostly studied in metastatic breast cancer patients, with numerous studies reporting that elevated CTC count, either at baseline or during or after treatment, is associated with a worse outcome [5–11]. The FDA-approved CellSearch detection system is currently the most frequently used system in the clinic for CTC detection, and randomized clinical trials are ongoing to establish utility [12]. In non-metastatic patients, the first results obtained with this system were generated in the REMAGUS02 study [13]: 23% of patients treated by neoadjuvant chemotherapy had ≥1 CTC detected in 7.5 mL of blood; CTC detection before and/or after neoadjuvant chemotherapy was interestingly not correlated with the primary tumor response to chemotherapy. This detection rate and the lack of association with tumor response were later confirmed by the GEPARQUATTRO study and other neoadjuvant studies [14, 15]. The REMAGUS02 study also reported that CTC detection before and/or after neoadjuvant chemotherapy had a negative impact on distant metastasis-free survival (DMFS; relative risk RR = 4.1, *P* = 0.013) after a short follow-up of 18 months [13]). At 3 years of median follow-up, we were able to confirm the impact of CTC detection before neoadjuvant chemotherapy on DMFS (RR = 5.0, *P* = 0.01) and also to detect a significant negative impact of CTC on overall survival (OS; RR = 9, *P* = 0.007) [16]. This article reports the updated results of the REMAGUS 02 CTC study, with a follow-up longer than 5 years.

## 2. Material and Methods

### 2.1. Patients and Treatments

All samples were obtained with the patient's written informed consent. The REMAGUS 02 trial was a prospective randomized multicentric trial conducted between 05/2004 and 10/2007, after approval by the regional ethics committee. Briefly, patients had stage II and III breast adenocarcinoma and were ineligible for breast conserving surgery at diagnosis. Patients received epirubicin-cyclophosphamide for 4 cycles followed by docetaxel for 4 cycles. HER2-positive patients were randomized to trastuzumab concomitant to docetaxel or no additional preoperative treatment. The main study results have been previously reported [17].

In an ancillary study, 118 patients had CTC count determined by the CellSearch system either before and/or after the neoadjuvant chemotherapy. All blood samples were obtained with the patient's written informed consent. Results of CTC analysis were available for 115 patients but were not disclosed to patients or clinicians. We previously reported the characteristics of patients included in this study: 45% had cT3 or cT4 breast cancers; 64% had loco-regional lymph node involvement; 48% had grade III breast cancers; hormone-receptors and HER2 were positive in 60% and 30% of cases, respectively. Overall pathological complete response (pCR) rate was 19% in this subgroup [13]. 

### 2.2. CTC Detection

CTC were detected by the CellSearch system (Veridex, Raritan, NJ), according to the manufacturer recommendations. This system has been described previously elsewhere [18]; the first step is an immunoenrichment procedure of EpCam-positive cells, followed by epithelial cytokeratin (+), DAPI (+), and CD45 (−) immunocytofluorescence. All CTC assessments were performed in a central laboratory (Institut Curie, Paris, France). We previously reported that ≥1 CTC/7.5 mL of blood (range of 0–17 CTC) were detected in 22 out of 97 patients screened at baseline (22%) and in 15 out of 86 patients screened at the end of chemotherapy (17%); CTC detection at inclusion and at the end of chemotherapy was not associated with any patients characteristics or with pathological complete response to chemotherapy (pCR) [13]. 

### 2.3. Statistical Analyses

DMFS and OS were defined as the time elapsed between the date of inclusion and the date of distant metastatic relapse or the date of death, respectively. Patients free of event were censored at the date of their last known contact. Survival and interval rates were calculated by the Kaplan-Meier method, and groups were compared using the log-rank test. Proportional hazards hypothesis was tested for each factor using Schoenfeld's residuals test and plotting. We introduced a time function in order to model that the hazard ratio evolved with the time. Cox proportional hazards model allowed introducing such factor with time-dependent effects. We tested four different functions relating hazard ratio to time: the linear function, square root function, square function and the log function. To select the most appropriate function, we minimized the Akaike information criterion (AIC). If the AIC of each function was very close, graphical adequacy was used to choose the time function. A significant level was fixed at 10% for univariate analysis using the Cox proportional hazards model. The selected covariates will be included in a multivariate analysis; we selected a multivariate model with a backward procedure with a significant level fixed at 5%. Multivariate analysis was carried out to assess the adjusted influence of prognostic factors using the Cox proportional hazards model. This report was written in accordance with the reporting of tumor marker studies guidelines [19].

## 3. Results

After updating patients' data, the median follow-up was 70 months (5.8 years) for the 115 patients included in this study. At time of analysis, 20 patients experienced a distant metastatic relapse (17%), and 14 died from their disease (12%). We did not observe nonbreast cancer-related death for all patients included in this study.

**Figure 1:**
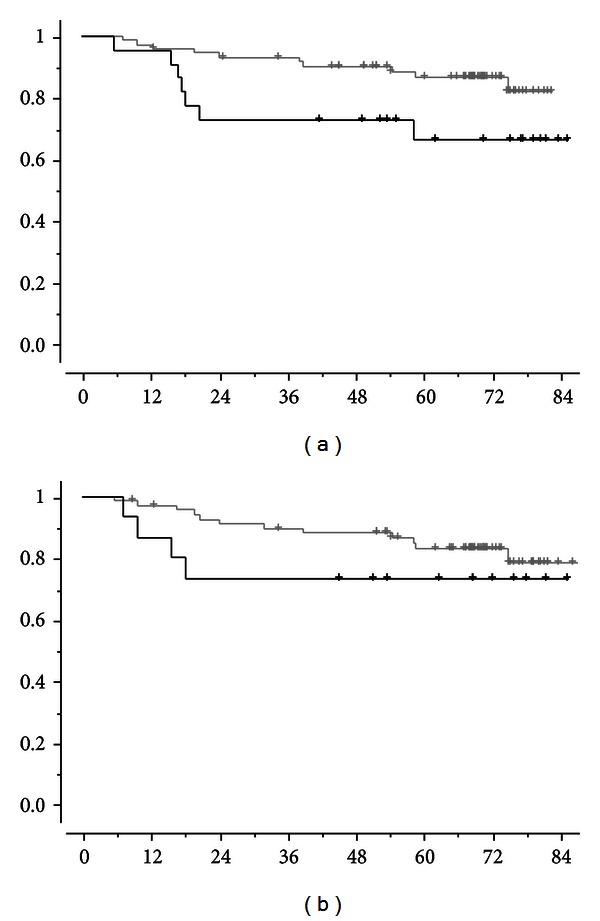
Distant metastasis-free survival, DMFS, in months, according to CTC detection before neoadjuvant chemotherapy ((a), *P* = 0.04) or after neoadjuvant chemotherapy ((b), *P* = 0.29). Black lines correspond to patients with ≥1 CTC/7.5 mL and grey lines to patients with no CTC detected.

**Figure 2:**
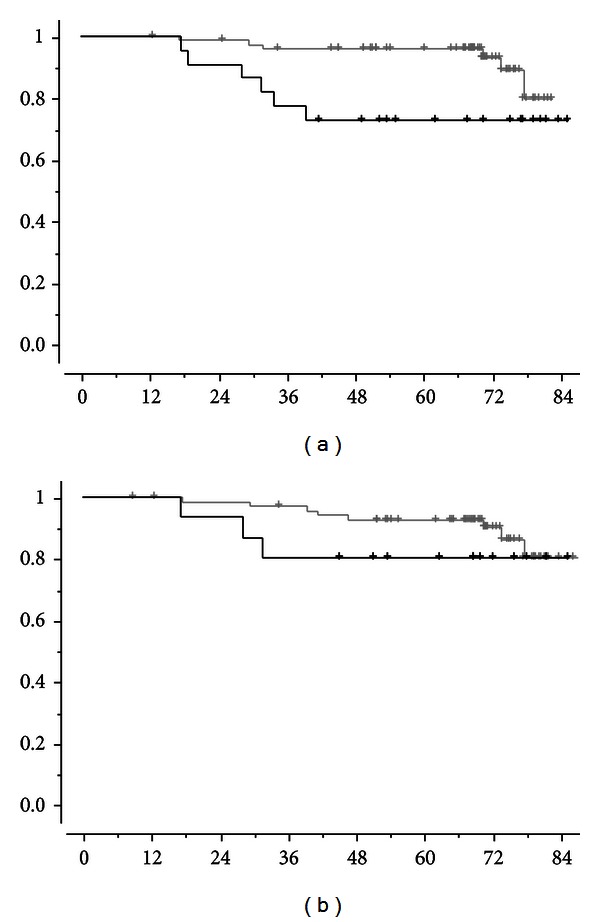
Overall survival, OS according to CTC detection before neoadjuvant chemotherapy ((a), *P* = 0.03) or after neoadjuvant chemotherapy ((b), *P* = 0.30). Black lines correspond to patients with ≥1 CTC/7.5 mL and grey lines to patients with no CTC detected.

DMFS according to prechemotherapy and postchemotherapy CTC count are shown in Figures 1(a) and 1(b), respectively: prechemotherapy detection of ≥1 CTC/7.5 mL was significantly associated with DMFS (*P* = 0.04), whereas postchemotherapy CTC detection had no significant impact. Similar findings were observed for OS: prechemotherapy and postchemotherapy CTC counts are shown in Figures 2(a) and 2(b), respectively.  Table 1 shows the impact of the other prognostic factor at univariate and multivariate analyses. As shown, pre-chemotherapy CTC and triple negative phenotype were the two independent prognostic factors for survival.

**Figure 3:**
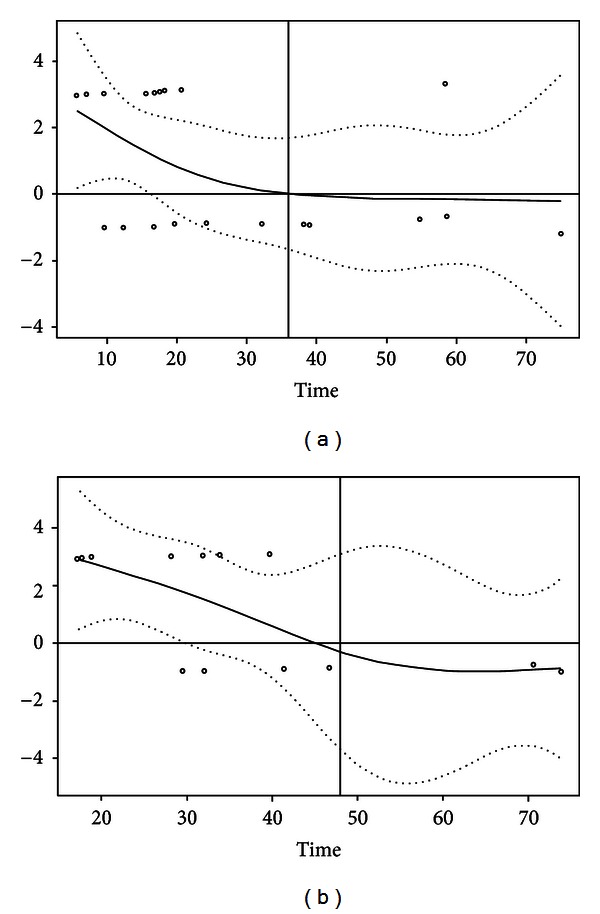
Hazard ratio estimates over time. The plain line shows the hazard ratio estimate (logarithmic scale) over time (in months) for DMFS (a) and OS (b), together with the 95% confidence interval (hashed lines). The vertical line is set at 36 months for DMFS and 48 months for OS. Circles show Schoenfeld's residuals.

Second, we investigated changes over time of the hazard ratio associated with CTC positivity (before and/or after the chemotherapy): Figure 3 shows that this hazard ratio (in logarithmic scale) decreased over time. For DMFS (Figure 3(a)), the hazard ratio decreased during the first 36 months and then was rather constant. However, the test for the proportional hazard assumption showed that the hazard did not significantly change over time for DMFS (*P* = 0.09). For OS (Figure 3(b)), the hazard ratio appeared to decrease to 1 (i.e., 0 in logarithmic scale) during the first 46 months, then became slightly negative (i.e., CTC associated with a better prognosis), and was then rather constant. The test for the proportional hazard assumption showed that the hazard significantly changed over time for OS (*P* = 0.02).

On these bases, we propose that CTC positivity was associated with a significantly worse outcome, for both DMFS (log rank: *P* = 0.007) and OS (log rank: *P* = 0.005), but mainly during the first 36 and 48 months of follow-up, respectively. For the patients who were still at risk (i.e., relapse-free) at these time points, CTC positivity had no clear prognostic impact on DMFS (log rank: *P* = 0.67) and OS (log rank: *P* = 0.42). We then introduced a time function in order to model the hazard ratio evolution over time of CTC detection. Linear time functions as well as square root functions, log functions, or simple polynomial functions were tried, but none generated reliable models for CTC impact on survival. 

## 4. Discussion

With now an extended median follow-up of 70 months, we confirm that the detection of ≥1 CTC/7.5 mL by the CellSearch system is associated with shorter DMFS and OS in breast cancer patients receiving neoadjuvant chemotherapy, despite the fact that this system may not detect cancer cells that have undergone epithelial-mesenchymal transition. Similar results have been obtained with other CTC detection techniques based on mRNA detection and that were independent of Ep-CAM expression. As shown by the multivariate analysis, this impact is independent of the other usual prognostic factors in the 115 patients included in this study. However, with an extended follow-up, we now report that the CTC detection impact on survival is lower than previously reported (hazard ratio (HR) = 9 after 36 months of follow-up; HR = 3 currently). Our results also suggest that CTC count before treatment may have a stronger impact on survival than CTC count after the completion of neoadjuvant chemotherapy. However, the incidence of CTC is lower in the posttreatment setting, which could reduce the power of our analysis. Looking at the actualized survival curves, we hypothesized that the impact of CTC detection on survival may be time dependent and possibly even limited to the first 3-4 years after the primary treatment. We have not been able to derive from our data a time function-based model that would significantly fit the results, but (i) only simple models were tested (ii) such models generally require many more patients. Recent results showing a prognostic value of CTC detection in the adjuvant setting in two large series were also published or presented with short follow-up around three years [20, 21], and analysis of more mature results will be of great interest. CTC number changes during treatment were not correlated with the outcome (data not shown), but no definite conclusion can be drawn, as these statistical analyses were clearly underpowered.

We believe that a longer follow-up of the REMAGUS02 CTC study will not make any significant impact on our original findings previously published, as survival data have now reached maturity. However, these data will be included in a planned metaanalysis of the studies that use the CellSearch technique in non-metastatic breast cancer setting. This would allow a more accurate comparison of prognostic significance of CTC detection before and after treatment.

## 5. Conclusion

With an extended median follow-up of 70 months, detection of ≥1 CTC/7.5 mL is still associated with shorter DMFS and OS in breast cancer patients receiving neoadjuvant chemotherapy. This factor's impact was mostly seen during the first three years of follow-up.

## Figures and Tables

**Table 1:** Univariate and multivariate analysis on survival.

Adverse prognostic factors	*N* patients/*N* total	DMFS univariate analysis *P* value	DMFS multivariate analysis RR, 95% CI, *P* value	OS univariate analysis *P* value	OS multivariate analysis RR, 95% CI, *P* value
Age ≤50 years	41/115	NS (0.99)	—	NS (0.51)	—
Tumor size T3 or T4	53/115	NS (0.13)	—	NS (0.28)	—
Clinical node N1 or N2	73/115	NS (0.64)	—	NS (0.49)	—
Tumor grade III	55/115	NS (0.76)	—	NS (0.55)	—
HR negative	47/115	NS (0.26)	—	0.01	Not included*
HER2 negative	81/115	NS (0.11)	—	0.04	Not included*
Triple negative phenotype	31/115	0.02	RR = 2.4 [0.9–6] *P* = 0.06	0.0003	RR = 5.4 [1.6–18] *P* = 0.006
Prechemotherapy ≥1 CTC/7.5 mL	22/95	0.04	RR = 2.4 [0.9–6] *P* = 0.06	0.03	RR = 3.0 [1.0–9.5] *P* = 0.05
Postchemotherapy ≥1 CTC/7.5 mL	15/85	NS (0.29)	—	NS (0.30)	—
Absence of pCR	93/114	NS (0.27)	—	NS (0.68)	—

DMFS: distant metastasis-free survival. OS: overall survival. 95% CI: 95% confidence interval. HR: hormone receptors. NS: nonsignificant. *HR and HER2 statuses, which are redundant to triple negative phenotype, were not included in multivariate analysis.

## References

[1] Bidard FC, Pierga JY, Soria JC, Thiery JP (2013). Translating metastasis-related biomarkers to the clinic—progress and pitfalls. *Nature Review Clinical Oncology*.

[2] Braun S, Vogl FD, Naume B (2005). A pooled analysis of bone marrow micrometastasis in breast cancer. *The New England Journal of Medicine*.

[3] Bidard FC, Vincent-Salomon A, Gomme S (2008). Disseminated tumor cells of breast cancer patients: a strong prognostic factor for distant and local relapse. *Clinical Cancer Research*.

[4] Janni W, Vogl FD, Wiedswang G (2011). Persistence of disseminated tumor cells in the bone marrow of breast cancer patients predicts increased risk for relapse—a European pooled analysis. *Clinical Cancer Research*.

[5] Cristofanilli M, Budd GT, Ellis MJ (2004). Circulating tumor cells, disease progression, and survival in metastatic breast cancer. *The New England Journal of Medicine*.

[6] Dawood S, Broglio K, Valero V (2008). Circulating tumor cells in metastatic breast cancer: from prognostic stratification to modification of the staging system?. *Cancer*.

[7] Nakamura S, Yagata H, Ohno S (2010). Multi-center study evaluating circulating tumor cells as a surrogate for response to treatment and overall survival in metastatic breast cancer. *Breast Cancer*.

[8] Nolé F, Munzone E, Zorzino L (2008). Variation of circulating tumor cell levels during treatment of metastatic breast cancer: prognostic and therapeutic implications. *Annals of Oncology*.

[9] Bidard FC, Mathiot C, Degeorges A (2010). Clinical value of circulating endothelial cells and circulating tumor cells in metastatic breast cancer patients treated first line with bevacizumab and chemotherapy. *Annals of Oncology*.

[10] Liu MC, Shields PG, Warren RD (2009). Circulating tumor cells: a useful predictor of treatment efficacy in metastatic breast cancer. *Journal of Clinical Oncology*.

[11] Pierga JY, Hajage D, Bachelot T (2012). High independent prognostic and predictive value of circulating tumor cells compared with serum tumor markers in a large prospective trial in first-line chemotherapy for metastatic breast cancer patients. *Annals of Oncology*.

[12] Bidard FC, Fehm T, Ignatiadis M (2013). Clinical application of circulating tumor cells in breast cancer: overview of the current interventional trials. *Cancer Metastasis Reviews*.

[13] Pierga JY, Bidard FC, Mathiot C (2008). Circulating tumor cell detection predicts early metastatic relapse after neoadjuvant chemotherapy in large operable and locally advanced breast cancer in a phase II randomized trial. *Clinical Cancer Research*.

[14] Riethdorf S, Müller V, Zhang L (2010). Detection and HER2 expression of circulating tumor cells: prospective monitoring in breast cancer patients treated in the neoadjuvant GeparQuattro trial. *Clinical Cancer Research*.

[15] Pierga JY, Petit T, Delozier T (2012). Neoadjuvant bevacizumab, trastuzumab, and chemotherapy for primary inflammatory HER2-positive breast cancer (BEVERLY-2): an open-label, single-arm phase 2 study. *The Lancet Oncology*.

[16] Bidard FC, Mathiot C, Delaloge S (2010). Single circulating tumor cell detection and overall survival in nonmetastatic breast cancer. *Annals of Oncology*.

[17] Pierga JY, Delaloge S, Espié M (2010). A multicenter randomized phase II study of sequential epirubicin/cyclophosphamide followed by docetaxel with or without celecoxib or trastuzumab according to HER2 status, as primary chemotherapy for localized invasive breast cancer patients. *Breast Cancer Research and Treatment*.

[18] Allard WJ, Matera J, Miller MC (2004). Tumor cells circulate in the peripheral blood of all major carcinomas but not in healthy subjects or patients with nonmalignant diseases. *Clinical Cancer Research*.

[19] McShane LM, Altman DG, Sauerbrei W (2005). Reporting recommendations for tumor marker prognostic studies (REMARK). *Journal of the National Cancer Institute*.

[20] Lucci A, Hall CS, Lodhi AK (2012). Circulating tumour cells in non-metastatic breast cancer: a prospective study. *The Lancet Oncology*.

[21] Rack B, Schindlbeck C, Andergassen U (2010). Prognostic relevance of circulating tumor cells in the peripheral blood of primary breast cancer patients. *Cancer Research*.

